# COVID-19 Surveillance in a Primary Care Sentinel Network: In-Pandemic Development of an Application Ontology

**DOI:** 10.2196/21434

**Published:** 2020-11-17

**Authors:** Simon de Lusignan, Harshana Liyanage, Dylan McGagh, Bhautesh Dinesh Jani, Jorgen Bauwens, Rachel Byford, Dai Evans, Tom Fahey, Trisha Greenhalgh, Nicholas Jones, Frances S Mair, Cecilia Okusi, Vaishnavi Parimalanathan, Jill P Pell, Julian Sherlock, Oscar Tamburis, Manasa Tripathy, Filipa Ferreira, John Williams, F D Richard Hobbs

**Affiliations:** 1 Nuffield Department of Primary Care Health Sciences University of Oxford Oxford United Kingdom; 2 General Practice and Primary Care Institute of Health and Wellbeing University of Glasgow Glasgow United Kingdom; 3 University Children's Hospital Basel University of Basel Basel Switzerland; 4 PRIMIS University of Nottingham Nottingham United Kingdom; 5 Department of General Practice Royal College of Surgeons, Ireland Dublin Ireland; 6 Department of Veterinary Medicine and Animal Productions University of Naples Federico II Naples Italy

**Keywords:** COVID-19, medical informatics, sentinel surveillance

## Abstract

**Background:**

Creating an ontology for COVID-19 surveillance should help ensure transparency and consistency. Ontologies formalize conceptualizations at either the domain or application level. Application ontologies cross domains and are specified through testable use cases. Our use case was an extension of the role of the Oxford Royal College of General Practitioners (RCGP) Research and Surveillance Centre (RSC) to monitor the current pandemic and become an in-pandemic research platform.

**Objective:**

This study aimed to develop an application ontology for COVID-19 that can be deployed across the various use-case domains of the RCGP RSC research and surveillance activities.

**Methods:**

We described our domain-specific use case. The actor was the RCGP RSC sentinel network, the system was the course of the COVID-19 pandemic, and the outcomes were the spread and effect of mitigation measures. We used our established 3-step method to develop the ontology, separating ontological concept development from code mapping and data extract validation. We developed a coding system–independent COVID-19 case identification algorithm. As there were no gold-standard pandemic surveillance ontologies, we conducted a rapid Delphi consensus exercise through the International Medical Informatics Association Primary Health Care Informatics working group and extended networks.

**Results:**

Our use-case domains included primary care, public health, virology, clinical research, and clinical informatics. Our ontology supported (1) case identification, microbiological sampling, and health outcomes at an individual practice and at the national level; (2) feedback through a dashboard; (3) a national observatory; (4) regular updates for Public Health England; and (5) transformation of a sentinel network into a trial platform. We have identified a total of 19,115 people with a definite COVID-19 status, 5226 probable cases, and 74,293 people with possible COVID-19, within the RCGP RSC network (N=5,370,225).

**Conclusions:**

The underpinning structure of our ontological approach has coped with multiple clinical coding challenges. At a time when there is uncertainty about international comparisons, clarity about the basis on which case definitions and outcomes are made from routine data is essential.

## Introduction

The COVID-19 pandemic has many features of a complex system [1,2]. Complexities include repeated name changes of both the causative organism and associated disease [3-6], evolving understanding of core clinical features at presentation [7,8], and differing rates of testing and approaches to outcome reporting between countries [9,10]. This complexity presents a significant challenge for consistent clinical coding within computerized medical records (CMR) systems [11].

Creating an ontology for COVID-19 surveillance should help to facilitate reproducibility and interoperability between various key stakeholders, from clinicians and epidemiologists to data scientists and software developers. Ontologies are formalizations of conceptualizations and exist in reference or application formats [12]. Reference ontologies are at a domain level and describe a group of related concepts. Application ontologies are more specific and are used when modeling across multiple domains [13]. Application ontologies should be evaluated against a testable use case, which represents the scope and requirements of the specific application [14,15]. The emergence of a new disease means that corresponding original ontologies need to be developed.

We report the development of a COVID-19 application ontology using the Oxford Royal College of General Practitioners (RCGP) Research and Surveillance Centre (RSC) network’s adaptations to COVID-19 as its use case. The RCGP RSC is an established primary care sentinel network, which extracts pseudonymized data from a nationally representative sample of over 500 general practices twice weekly (N=5,370,225) [16]. RCGP RSC has collaborated with Public Health England (PHE) for over 50 years, conducting influenza and respiratory disease surveillance and vaccine effectiveness studies [17,18]. The RCGP RSC has extended these routine surveillance activities to include monitoring the spread of COVID-19, assessing the effectiveness of containment measures, and becoming a platform for an in-pandemic COVID-19 trial [19,20].

Building on our previous experience of developing ontologies [12,21,22], we created an application ontology for extended COVID-19 surveillance.

## Methods

### Overview

Our application ontology was developed in 3 stages—stage 1: creating and testing our use case; stage 2: developing the COVID-19 surveillance ontology; and stage 3: external validation using a rapid Delphi consensus exercise. We classified this as an application ontology because the system crosses a range of domains to deliver specific goals. The data source was routine primary care data from the RCGP RSC sentinel network, combined with virology and serology sampling data from PHE. Additionally, a practice dashboard and a practice liaison team helped ensure data quality [23]. Episode type, whether a case is a first or incident case or a follow-up is important for surveillance; while we can infer this from records it is better if it is collected as primary information [24].

### Stage 1: Creating and Testing the Use Case

We created a testable narrative use case for COVID-19 surveillance, using previously described methods [25,26]**.** The primary actor was the RCGP RSC, the system it interacts with was the national response to the COVID-19 pandemic, and its outcomes entailed monitoring spread and effect of mitigation measures.

The use case has been progressively implemented in-pandemic. We report our implementation across the domains identified. As our ontology developed and formalized, post hoc checking was done to ensure extracts were ontology compliant.

### Stage 2: Developing the COVID-19 Surveillance Ontology

We developed an application ontology to support extended surveillance using routine CMR data. The terminology and clinical understanding and response to COVID-19 were rapidly changing during the development period. The ontology has built-in flexibility to accommodate these and further changes.

We used our 3-step ontological process to identify codes to meet our requirements [12,21,22]:

- Step 1: the ontology layer; defines relevant COVID-19 surveillance concepts and may include exposure, investigations, diagnoses, or other “processes of care.” Part of our ontological process is to iterate whether cases identified are definite, probable, or possible, based on the specificity of the codes used (an approach developed in diabetes research [27]);
- Step 2: the coding layer; applies concepts of the ontology layer to the specific coding system used in the CMR. Individual codes are classified as having direct, partial, or no clear mapping to the criteria considered [28]. In this case, we extended this to exclude suspected cases where there was a subsequent negative test. Post hoc data validation was done largely via our practice liaison team. One member of the team was entirely dedicated to ensuring data quality providing anticipatory training and coding aids, and a responsive service;
- Step 3: the logical data extract model; systematically tests the codes identified to ensure that data outputs are consistent with study requirements.

We wanted our resource to be findable, accessible, interoperable, and reusable (FAIR) [29], so we used standard tools in its development, namely the Protégé ontology development environment [30] and Web Ontology Language (OWL) [31].

The scope of the ontology included:

- Demographic details, including age, gender, ethnicity, deprivation, rurality, and linking key identifiers;
- Recording of monitored conditions and key clinical features (ie, symptoms and signs);
- Relevant comorbidities and risk factors;
- Tests and test results (ie, COVID-19–specific and test results that might imply susceptibility or resilience);
- Key outcome measures including hospitalization, oxygen therapy, intensive care admission, and mortality.

### Stage 3: External Evaluation of the Ontology

We carried out a rapid Delphi consensus exercise by inviting a panel (n=9) of international primary care clinicians and informaticians through the International Medical Informatics Association Primary Health Care Informatics working group and extended networks [32,33]. The consensus exercise consisted of 3 rounds:

1. We shared our initial ontology and requested panel members to inform us about additional concepts that were not present in the ontology but present in their clinical workflows. In order to facilitate rapid consensus, we used email correspondence for this stage.
2. We shared the revised ontology with panel members, who were asked to indicate their level of agreement, on a 5-point Likert scale, to statements related to the coverage of concepts and applicability of the ontology to their primary care system. This was delivered through on an online survey (see panel members and questions in Multimedia Appendix 1). Consensus was defined as ≥80% agreement. Statements not meeting 80% agreement were modified according to the feedback provided by the expert panel and redistributed to panelists for round 3.
3. We conducted an online discussion to review and approve the final ontology.

### Ethical Considerations

COVID-19 surveillance is carried out by RCGP RSC in collaboration with PHE, and approved under Regulation 3 of The Health Service (Control of Patient Information) Regulations 2002 by PHE’s Caldicott Guardian [34]. No specific permissions were needed for our ontology development as no additional processing of data was required.

## Results

### Stage 1: Creating and Testing the Use Case

We developed a summary narrative use case (Table 1). The success scenarios listed are goals we want to achieve.

The success scenarios and extensions reflect the cross-domain activities within the use case. We list the outcomes across 5 domains: primary care, public health, virology, clinical research, and clinical informatics (Table 2). We implemented our ontology through practical activities across these domains.

**Table 1:** Summary narrative use case.

Variable		Description
Actor		Oxford Royal College of General Practitioners Research and Surveillance Centre
Scope		Delivery of COVID-19 surveillance and research
Level		Health care system wide
**Stakeholders and interests**		
	Patients and public	Safe and timely guidance through the pandemic
	General practices	Professional interest; payment; providing high-quality, evidence-based care
	Public Health England	Need data to predict transmission Monitor the effectiveness of interventions
	Royal College of General Practitioners	Care for/protect members Contribute to pandemic response
	Primary care clinical trials unit	Data governance policies control which data can be viewed Recruit to trial to mitigate COVID-19
Precondition		Legal basis, permissions for data extracts, data extraction, and analytics capability within the network
Minimal guarantee		Delivery of data and analytics at prepandemic scale
Success guarantee		Larger network with high-quality data Outputs to meet changed requirements during the pandemic Authoritative source of primary care data, evidenced by academic publication
Main success scenario		High-quality primary care data, feedback to practices via customized dashboards Representative sampling of virology and serology by collecting the specified number of samples (900 virology, 1000 serology per week) Twice weekly data feeds to Public Health England to meet their data requirements National observatories and weekly return that represent the impact of COVID-19 Ensure that we fully recruit to the PRINCIPLE^a^ and other trials through the Oxford–RCGP RSC system High-quality publication of lessons from surveillance
Extensions		Trebling the number of virology practices (we have gone from 100 to 300 virology sampling practices, from 10 to 200 serology sampling practices) Adjusting to the effect of lockdowns on: Extending the network to over 1000 practices to support large-scale clinical trials, embedded in clinical practice; eg, recruitment into the PRINCIPLE trial Sampling all eligible patients due to the reduced number seen on surgery premises Postconvalescent serology; we will collect convalescent serology at 28 days from a wide range of practices Managing unforeseen problems: Refusal of some post offices to allow sample postage Postage delays Swab supply problems Piloting new methods of swab delivery to patients Add resilience to the surveillance system Human resilience - extending data team and support System resilience - direct feeds from major CMR^b^ suppliers Other studies: large numbers of study requests that need managing

^a^PRINCIPLE: Platform Randomised Trial of Interventions Against COVID-19 in Older People.

^b^CMR: computerized medical record.

**Table 2:** Application use-case outcomes by domain.

Domain	Description	Outcomes
Primary care	COVID-19 Observatory - temporal and geographic surveillance COVID-19 dashboard - practice-level data quality	Data quality feedback to practices Feedback from practices
Public health	COVID-19 - supplementary report Public health policy - containment measures	Trends of community transmission after social distancing ends Estimates of COVID-19–related community morbidity and mortality
Virology	Swabbing - investigation Virology Serology	Virologically confirmed incidence Representative collection of serology for sero-epidemiology Ordering stock control and swab and virology container supply
Clinical research	Recruitment to clinical trials	Health outcomes: chest infections, hospitalization, intensive care unit, mechanical ventilation, oxygen therapy, and death
Clinical informatics	IG^a^—legal basis, data sharing agreements, contracts Hardware and its resilience Semantic interoperability across domains	Data quality, usability, FAQs^b^—continuous improvement of our interface Adaptability with changing clinical knowledge Ontology with annotations to clinical terms/codes

^a^IG: information governance.

^b^FAQ: frequently asked question.

#### Primary Care Domain: Data Quality and Feedback to General Practices (COVID-19 Dashboard)

**Figure 1:**
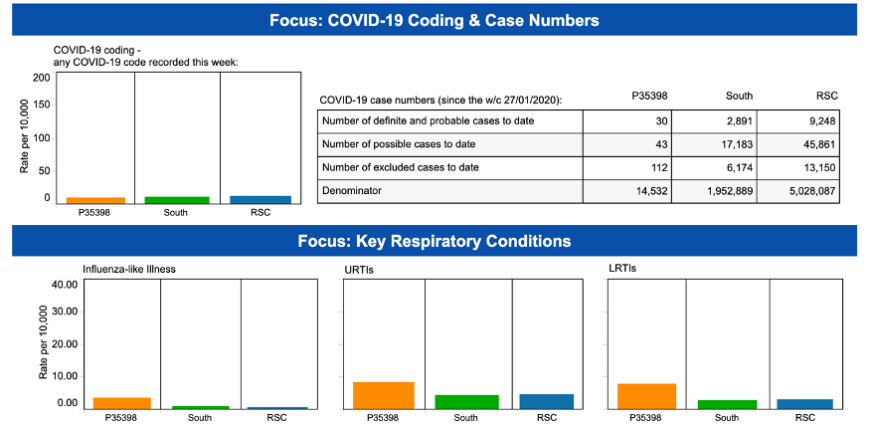
COVID-19 dashboard for each RCGP RSC network practice [36]. The column starting P35398 is that practice data; “South” is their region; RSC is the rate across the whole network. Dates are presented in the DD/MM/YYYY format throughout. RCGP RSC: Oxford Royal College of General Practitioners (RCGP) Research and Surveillance Centre; URTI: upper respiratory infection; LRTI: lower respiratory infection.

Our COVID-19 dashboard presented weekly data on respiratory conditions to practices within the RCGP RSC sentinel network. Data were presented on COVID-19 incidence for the individual practice, and at the regional and national levels for reference, along with rates of other respiratory infections (Figure 1). Postimplementation feedback had to keep pace with multiple data changes and different timetables of code releases between CMR system providers. It included constant updating of coding prompt cards [35]. It also required liaison with computer template developers to change design to incorporate episode type.

#### Public Health Domain: Data Visualization With COVID-19 Observatory

**Figure 2:**
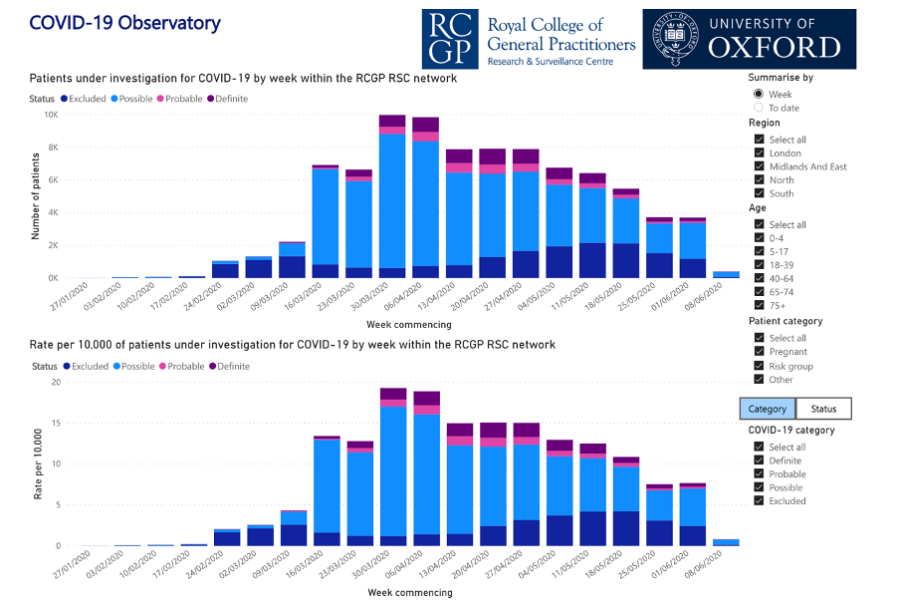
Oxford RCGP RSC interactive COVID-19 observatory. Users can select the cumulative or week-by-week view of the data, and visualize data by age-band, region, risk group, and COVID-19 status (definite, probable, possible, and excluded) [37]. RCGP RSC: Oxford Royal College of General Practitioners Research and Surveillance Centre.

Our ontology ensured consistency between our classic weekly return, which now includes COVID-19 surveillance. In addition, we developed customized outputs for epidemiologists at PHE and an observatory to present data on the incidence of COVID-19 across the network (Figure 2). This is based on coding described in the ontological layer and presents incidence rate per 10,000 cases of COVID-19. Up to the week commencing September 21, 2020, we have identified a total of 19,115 people with definite COVID-19, 5226 probable cases, and 74,293 people with possible COVID-19 within the RCGP RSC network (N=5,370,225).

The biggest area of challenge was attribution of codes to certainty of diagnosis. We have had to evolve this with coding system changes (see Multimedia Appendix 1 for our final SNOMED CT [SNOMED Clinical Terms] concept list).

#### Virology Domain: Weekly Virologic Surveillance Reports

**Figure 3:**
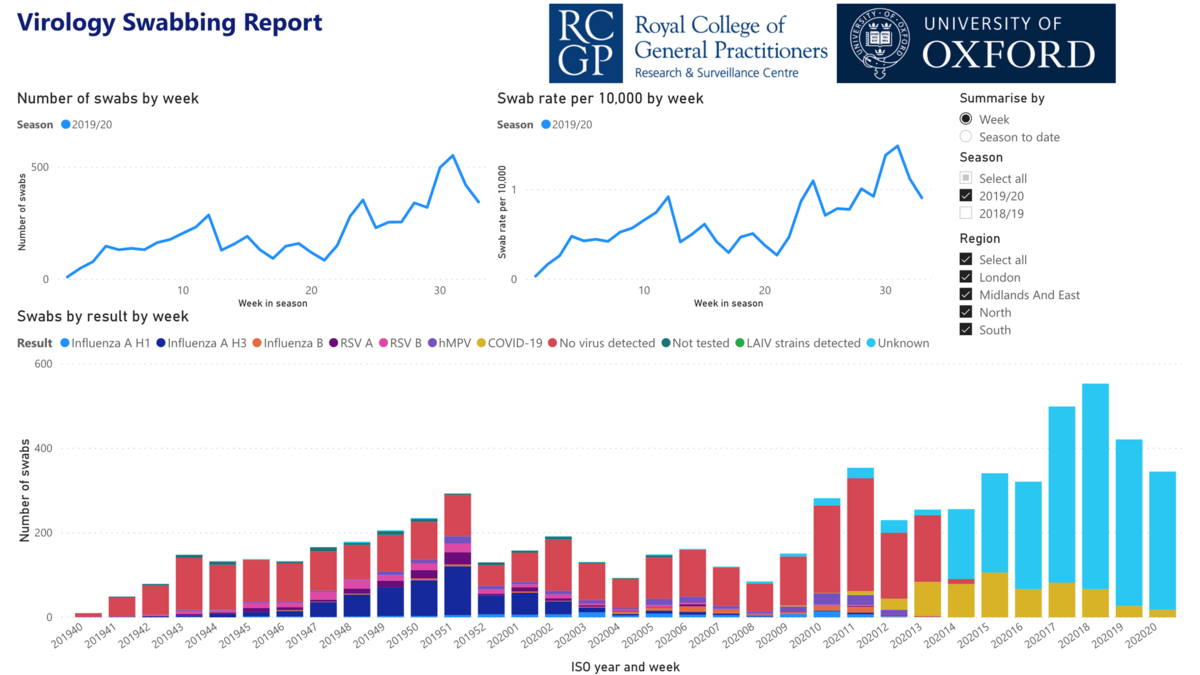
Oxford RCGP RSC interactive virology swabbing report. Users can look at the cumulative or weekly report or compare with the previous year, and look by infected organism or region. “Unknown” is used where no testing is done; currently, samples are only tested for COVID-19. RCGP RSC: Oxford Royal College of General Practitioners Research and Surveillance Centre; ISO: International Organization for Standardization.

Similarly, our ontology drove the consistent extension of our virology reporting. Sound data structures have also been important because the number of participating virology sampling practices trebled from 100 to 300 to provide more data. The weekly virology report provides a visualization of the absolute number and rate per 10,000 by week of the swabs taken, combined with the matched week from the previous year’s figures for background context (Figure 3). There is a similar observatory for serology (included in Multimedia Appendix 1).

#### Clinical Research Domain: Participation in Observational and Interventional Studies

The COVID-19 surveillance application ontology supported consistent reporting of findings in observational and interventional clinical research. We have a series of ongoing observational studies, the first of which has reported results [38]. The network is also supporting the PRINCIPLE (Platform Randomised Trial of Interventions Against COVID-19 in Older People) trial, a UK platform randomized controlled trial of interventions for COVID-19 in primary care. The study is assessing the effectiveness of trial treatments in reducing the need for hospital admission and death in patients with suspected COVID-19 infection aged ≥50 years with serious comorbidity, and aged ≥65 years with or without comorbidity [20]. To date, 830 practices have signed up, with 415 patients randomized; 468 (56.4%) of these are RCGP RSC practices, and they have recruited 342 (82.4%) of the included patients so far.

#### Clinical Informatics Domain: Creating the COVID-19 Ontology

The annotated application ontology was published on the BioPortal Ontology Repository [39] and will continue to be developed as our understanding of COVID-19 advances and new interventions (eg, vaccination) are introduced. The detail of the ontological development is set out in stage 2 of our 3-step process.

### Stage 2: Developing the COVID-19 Surveillance Ontology

#### Step 1: Ontological Layer

We reviewed emerging case definitions of COVID-19 to identify key concepts used for case ascertainment and their relationships. Concepts included in the ontology were consistent with the WHO data dictionary for COVID-19 case–based reporting [40].

**Figure 4:**
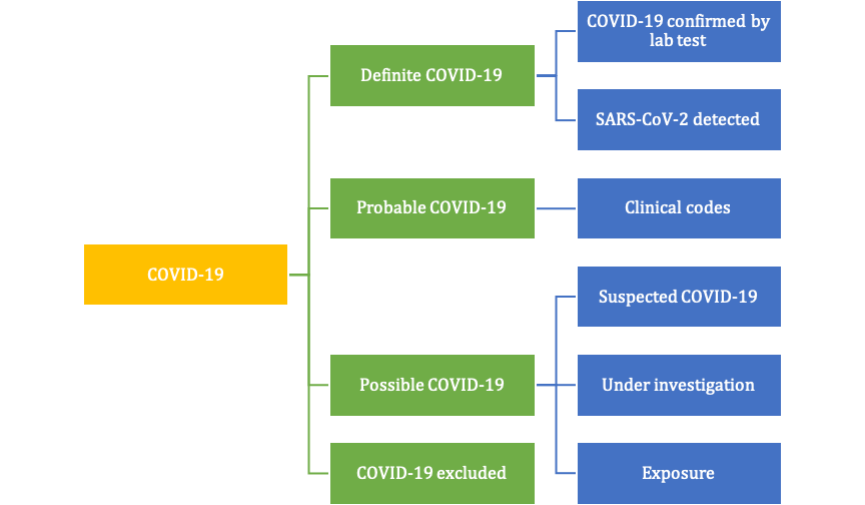
Foundational ontological concepts used for COVID-19 surveillance.

We have limited our presentation of results to the case definition of COVID-19. This has involved grouping concepts into: (1) definite, which include definitive codes for a laboratory-confirmed case of COVID-19; (2) probable, which included a clinical diagnosis of COVID-19 and use of out-of-date codes created during the previous SARS (severe acute respiratory syndrome) outbreak; (3) possible, which contains a range of coding alternatives related to suspected COVID-19 investigation but no result and exposure codes; and (4) excluded, where a test requested is reported as negative (this is demonstrated in Figure 4). At the individual level, the tests work hierarchically, with the most specific one driving the categorization.

#### Step 2: Coding Layer

We completed a dynamic process of mapping clinical terminology codes to concepts that emerged from our ontological layer (Table 3).

The National Health Service (NHS) uses the UK SNOMED CT system of coding, which is normally only updated twice yearly. In early February 2020, there were no clinical codes specific to COVID-19. Initially, CMR suppliers created 5 new system-wide local codes to support essential COVID-19–related recording within a week of being requested [11,19]. Subsequently, 2 emergency releases of novel COVID-19–related UK SNOMED CT codes were developed through a rapid consultation process conducted by the NHS Digital Information Representation Service [41], as greater clinical insight into COVID-19 and stability around nomenclature emerged. These UK SNOMED CT concepts were developed independently of international SNOMED CT terminology development; however, this open-source ontology can be mapped to international terms with ease. We iteratively annotated the ontological concepts with these stepwise-released COVID-19 SNOMED CT clinical concepts.

**Table 3:** Migration across SNOMED CT (SNOMED Clinical Terms) concepts released from February to May 2020.

Clinical concepts that should be coded in CMR^a,b^		Temporary codes^c^	Final SNOMED CT description
**COVID-19 definite**		Confirmed 2019 nCoV (Wuhan) infection OR Confirmed 2019 nCoV (novel coronavirus) infection	COVID-19 confirmed by laboratory test SARS-CoV-2 (severe acute respiratory syndrome coronavirus 2) detected
**COVID-19 probable**		No specific codes	COVID-19 COVID-19 confirmed by clinical diagnostic criteria
**COVID-19 possible**			
	Exposure to infectious agent	Exposure to 2019 nCoV (Wuhan) infection OR Exposure to 2019 nCoV (novel coronavirus) infection	Exposure to SARS-CoV-2 (severe acute respiratory syndrome coronavirus 2) infection
	Suspected infection	Suspected 2019 nCoV (Wuhan) infection OR Suspected 2019 nCoV (novel coronavirus) infection	Suspected COVID-19
	Test for infectious agent offered or taken	No specific codes	Swab for SARS-CoV-2 (severe acute respiratory syndrome coronavirus 2) taken by health care professional Self-taken swab for SARS-CoV-2 (severe acute respiratory syndrome coronavirus 2) offered Self-taken swab for SARS-CoV-2 (severe acute respiratory syndrome coronavirus 2) completed
		Tested for 2019 nCoV (Wuhan) infection OR Tested for 2019 nCoV (novel coronavirus) infection	—^d^
**COVID-19 excluded**		Excluded 2019 nCoV (Wuhan) infection OR Excluded 2019 nCoV (novel coronavirus) infection	COVID-19 excluded COVID-19 excluded by laboratory test SARS-CoV-2 (severe acute respiratory syndrome coronavirus 2) not detected

^a^CMR: computerized medical record.

^b^From ontological layer.

^c^Used until replacement with SARS-CoV-2/COVID-19 concepts.

^d^Not applicable.

#### Step 3: Logical Data Extract Layer

**Figure 5:**
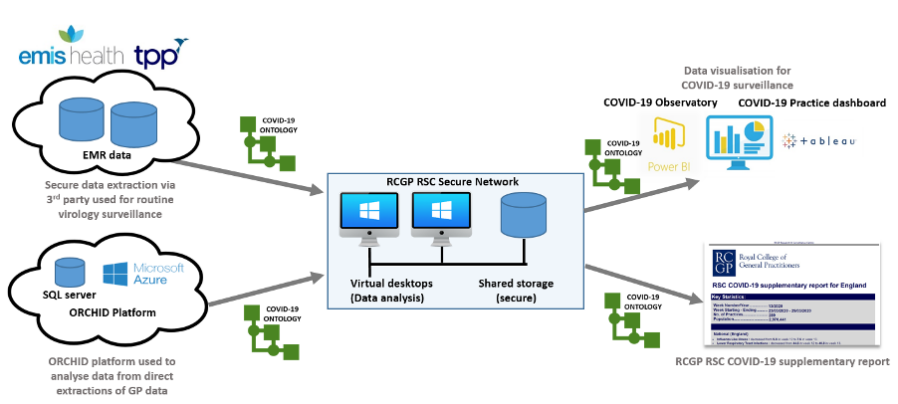
Use of the COVID-19 surveillance ontology across the RCGP RSC processes to achieve semantic consistency in data extraction, visualizations, and surveillance reports. EMR: electronic medical record; GP: general practitioner; ORCHID: Oxford RCGP Clinical Informatics Digital Hub; RCGP RSC: Oxford Royal College of General Practitioners Research and Surveillance Centre; SQL: Structured Query Language.

We incorporated the annotated ontology into the routine surveillance platform of the RCGP RSC data. The ontology identified various states of COVID-19 diagnosis in the incoming data feeds used for surveillance. We conducted a week-by-week analysis of incoming data modifying our outputs to take account of supplier-specific changes in reporting. We are planning for cloud-based extracts and customized extracts from individual CMR vendors; to do this we are creating an Oxford RCGP Clinical Informatics Digital Hub (ORCHID) (Figure 5) [42].

#### External Evaluation of the Ontology

While we obtained a good consensus in our Delphi exercise, there was important learning and priorities flagged for development. Consensus was obtained for 7 out of 8 (87.5%) of the statements related to coverage of concepts under the upper level headings of the COVID-19 ontology. All panel members except one agreed with statements relating to the applicability of the ontology for case finding activities in their local primary care setting (Table 4). Input from panel members guided expansion of the concepts related to statements not reaching consensus, and this was reviewed by panel members in round 3 of the Delphi exercise.

**Table 4:** Number of responses and % agreement (strongly agree/agree) to statements relating to the applicability of the ontology for case finding activities in panel members’ local primary care setting.

Statement		Strongly disagree, n	Disagree, n	Neither agree or disagree, n	Agree, n	Strongly agree, n	% Agreement
**Please indicate your level of agreement with the coverage of concepts given under each upper level heading of the COVID-19 surveillance ontology.**							
	Symptoms and signs	0	1	0	3	5	88.9
	Past medical history/at-risk conditions	0	0	3	2	4	66.7
	Exposure	0	0	0	2	7	100
	Investigations	0	0	0	5	4	100
	COVID-19 case status	0	0	0	1	8	100
	Interventions	0	1	0	3	5	88.9
	Process of care	0	0	1	1	7	88.9
	Outcomes	0	0	0	5	4	100
The COVID-19 ontology in its current format is suitable for COVID-19 case ascertainment in my local primary care setting.		0	0	1	5	3	88.9

Softer important discussion points emerged; for example, our symptom collection is relatively poorly developed and that there remains uncertainty about risk and protective factors. There was strong feeling among one expert that vaccination and exposures should be part of the ontology; these were subsequently added.

## Discussion

### Principal Findings

We rapidly developed an application ontology in-pandemic to support extended surveillance and research activities across the 5 clinical and informatics domains described in our use case. This application ontology has provided a framework, which we have used to help ensure the reliability and consistency of our outputs at a time of change. This iterative ontological approach is flexible and robust enough to match the pace and direction of the evolving clinical landscape of COVID-19.

The focus of our work has been on case identification and associated test results, as these are the foundations on which epidemiological and interventional studies are based. We felt it appropriate to flag the certainty with which a diagnosis is made. We have already used this ontology in observational and interventional studies [20,38].

The separation of the coding layer from the ontological (conceptual) layer allows surveillance to be resilient while new case definitions and clinical codes are added to general practice CMR systems. This approach ensures transparency in case definitions used for reporting and facilitates clear communication by allowing clinicians, database developers (involved in extracting data from practices’ data sources), and practice liaison officers (who advise practices about data recording best practices) to maintain consistency within an organization.

This application ontology could easily and rapidly be adapted for COVID-19 surveillance and clinical research in various other countries and health care networks. As the COVID-19 pandemic continues, there is enormous global pressure on health care systems to understand trends in incidence rates and conduct high-quality research; this ontology is open-source and can be mapped onto local clinical coding systems to permit consistency in analyses.

### Comparison With Previous Literature

To our knowledge, this is the first time that a systematic ontological approach has been developed in-pandemic for extended disease surveillance, using structured routine clinical data. This application ontology aligns with previous clinical informatics literature on application ontology engineering and validation through the testable use-case approach [43,44].

There are other pandemic surveillance systems that look at open-source, unstructured data, such as media reports and clusters of symptom-related internet searches, extracting information of epidemiological relevance [45]. Examples of such systems include BioCaster [46], the Global Public Health Intelligence Network [47], ProMed [48], and HealthMap [49]. The latter three systems are working under the WHO collaborative, the Epidemic Intelligence from Open Sources initiative, which played a role in the identification of the COVID-19 outbreak from early media reports from China in December 2019 [50]. Some of the event-based pandemic surveillance systems have published ontological foundations in the public health and surveillance domains [46,51,52]. While useful for providing supplementary information to epidemiologists on the emergence of an outbreak in real time, these knowledge representations do not specifically address the types of information described in clinical data, such as presenting complaint, comorbidities, virology, or health outcomes.

There are very limited studies of data platforms’ performance within integrated clinical surveillance systems [45]. The lack of accurate and available data to underpin epidemic forecasting in emerging outbreaks has been highlighted [53].

We found no literature using an ontological approach for COVID-19 surveillance. There are domain ontologies related to the coronavirus published on BioPortal. The first focuses on the wider *Coronaviridae* family and their biochemical and microbiological properties [54], while the second was developed to provide semantic assistance for clinical research form completion [55]. None were designed to integrate the various clinical data streams necessary to carry out COVID-19 surveillance.

### Strengths and Limitations

The 3-step iterative ontological process that we have implemented has proven to be suitably flexible to cope with the changes in COVID-19 terminology and CMR system codes. A further strength was the implementation and deployment of this ontology, considering the FAIR guiding principles [29]. The ontology is discoverable and accessible on the BioPortal ontology repository. This application ontology, built using best practices around defining and testing a use case, is inherently interoperable and reusable [29]. In the absence of a gold-standard infectious disease surveillance ontology, we believe our attempts at achieving a degree of consensus and external validity from a range of international experts in the field of clinical informatics and primary care as a major strength of the current study. While the Delphi panel size was relatively small and a limitation, we purposefully selected panel members from a range of countries with varied clinical coding systems.

We focused on case finding and results; we now need to turn our attention to presenting symptoms, particularly looking to focus on those that may be of prognostic value and emerging treatments including vaccination. Our ontology as currently run will classify false positive lab results incorrectly, and we recognize this is a limitation that should be noted by users. Additional limitations were its development in a single sentinel system and that it was not developed ready to integrate into a common data model [56].

### Conclusions

We have created a COVID-19 application ontology, with strengths that include its speed of development, being openly shared via BioPortal, and its adaptability. The limitations are its development in a single sentinel network and its current limited focus. The ontology should make conclusions based on primary care sentinel data more transparent and facilitate pooled analyses in COVID-19 surveillance and research. We welcome any requests for information on applying our COVID-19 surveillance application ontology to other health care settings, both domestically and internationally.

## Appendix

Multimedia Appendix 1 Supplementary tables and figures.

## Abbreviations

CMR: computerized medical record
FAIR: findable, accessible, interoperable, and reusable
NHS: National Health Service
OWL: Web Ontology Language
PHE: Public Health England
PRINCIPLE: Platform Randomised Trial of Interventions Against COVID-19 in Older People
RCGP: Oxford Royal College of General Practitioners
RSC: Research and Surveillance Centre
SARS: severe acute respiratory syndrome
SNOMED CT: SNOMED Clinical Terms
ORCHID: Oxford RCGP Clinical Informatics Digital Hub
