# Supplementary material for: COVID-19 Surveillance in a Primary Care Sentinel Network: In-Pandemic Development of an Application Ontology
Source: JMIR Public Health Surveill. 2020 Nov 17;6(4):e21434. doi: 10.2196/21434 (PMC7674143; doi:10.2196/21434)
Supplement: Multimedia Appendix 1 [file publichealth_v6i4e21434_app1.docx]

**Supplementary Material**

**In-pandemic development of an application ontology for COVID-19 surveillance in a primary care sentinel network.**

**
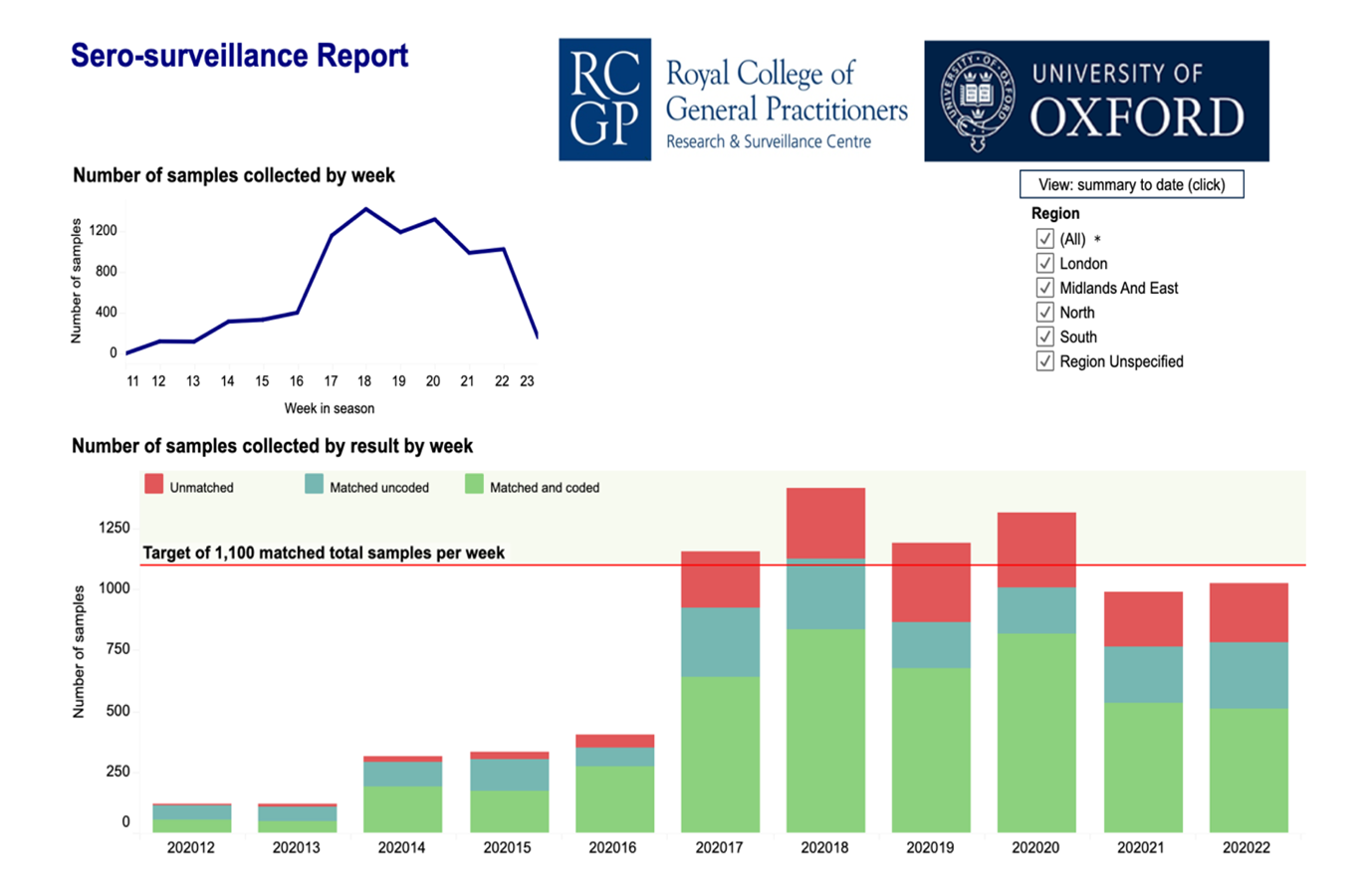
**

**S1: Oxford RCGP RSC interactive serology sampling dashboard (**[**link**](https://clininf.eu/index.php/serology/)**)**

Users can select the cumulative or week-by-week view of data on samples collected for serological investigation, and visualise data by age-band and region.

**S2: – Complete COVID-19 Surveillance Application ontology**

***Table 1.* SNOMED CT concepts for symptoms and signs from COVID-19 surveillance ontology.**

|  | **Semantic tag** | **ConceptID** | **Term** |
| --- | --- | --- | --- |
| **Symptoms** |  |  |  |
|  | Finding | 386661006 | Fever |
|  | Finding | 49727002 | Cough |
|  | Finding | 297216006 | Increasing breathlessness |
|  | Finding | 230145002 | Difficulty breathing |
|  | Finding | 267036007 | SOB - Shortness of breath |
|  | Finding | 55929007 | Feeling irritable |
|  | Finding | 26544005 | Muscle weakness |
|  | Finding | 267102003 | Sore throat symptom |
|  | Finding | 82297005 | Sinus congestion |
|  | Finding | 267060006 | Diarrhoea symptom |
|  | Finding | 272044004 | Complaining of vomiting |
|  | Finding | 22253000 | Pain |
|  | Finding | 82991003 | Generalised aches and pains |
|  | Finding | 29857009 | Chest pain |
|  | Finding | 21522001 | Abdominal pain |
|  | Finding | 271801002 | Taste sense altered |
|  | Finding | 83156004 | Sense of smell impaired |
|  | Finding | 56018004 | Wheeze |
|  | Finding | 272040008 | Wheezing symptom |
|  |  |  |  |
| **Signs** |  |  |  |
|  | Property | 722490005 | Temperature |
|  | Finding | 164303007 | O/E - temperature elevated |
|  | Finding | 271897009 | On examination - fever |
|  | Finding | 386661006 | Fever |
|  | Finding | 386661006 | Pyrexia |
|  | Finding | 386661006 | Febrile |
|  | Finding | 162916002 | O/E - tachypnoea |
|  | Finding | 49436004 | Atrial fibrillation |
|  | Finding | 3424008 | Tachycardia |
|  | Finding | 48867003 | Bradycardia |
|  | Finding | 1066831000000104 | Atrial fibrillation detected |
|  | Finding | 57948007 | Agnosia for taste |
|  | Finding | 164086009 | O/E - anosmia |
|  | Finding | 162894004 | O/E - expiratory wheeze |
|  | Finding | 704114003 | On examination - inspiratory wheeze |
|  |  |  |  |

***Table 2.* SNOMED CT concepts for co-morbidities and at risk conditions from COVID-19 surveillance ontology.**

|  | **Semantic tag** | **ConceptID** | **Term** | **Semantic tag** | **ConceptID** | **Term** |
| --- | --- | --- | --- | --- | --- | --- |
| **Co-morbidities/** |  |  |  |  |  |  |
| **At-risk**  **conditions** | Finding | 77386006 | Pregnant |  |  |  |
|  |  |  |  | Qualifier value | 255246003 | First trimester |
|  |  |  |  | Qualifier value | 255247007 | Second trimester |
|  |  |  |  | Qualifier value | 255248002 | Third trimester |
|  | Qualifier value | 255410009 | Postpartum |  |  |  |
|  |  |  |  |  |  |  |
|  | Disorder | 234532001 | Immunodeficiency |  |  |  |
|  |  |  |  | Disorder | 58606001 | Primary immune deficiency  disorder |
|  |  |  |  | Disorder | 33286000 | Secondary immune deficiency  disorder |
|  |  |  |  |  |  |  |
|  | Disorder | 49601007 | CVD |  |  |  |
|  |  |  |  | Disorder | 38341003 | Hypertension |
|  |  |  |  | Disorder | 84114007 | Heart failure |
|  |  |  |  | Disorder | 698247007 | Arrhythmia |
|  |  |  |  | Disorder | 414545008 | Ischemic heart disease |
|  | Disorder | 73211009 | Diabetes |  |  |  |
|  |  |  |  |  |  |  |
|  | Disorder | 709044004 | CKD |  |  |  |
|  |  |  |  | Disorder | 90708001 | Kidney disease |
|  |  |  |  | Disorder | 90688005 | Chronic renal failure  syndrome |
|  |  |  |  |  |  |  |
|  | Disorder | 328383001 | Chronic liver disease |  |  |  |
|  |  |  |  |  |  |  |
|  | Disorder | 363346000 | Neoplasm |  |  |  |
|  |  |  |  | Disorder | 55342001 | Neoplasm |
|  |  |  |  | Disorder | 372087000 | Primary malignant  neoplasm |
|  |  |  |  | Disorder | 93143009 | Leukaemia |
|  |  |  |  | Disorder | 118600007 | Lymphoma |
|  |  |  |  | Disorder | 415110002 | Plasma cell myeloma |
|  |  |  |  | Disorder | 109989006 | Multiple myeloma |
|  |  |  |  |  |  |  |
|  | Disorder | 413839001 | Chronic lung disease |  |  |  |
|  |  |  |  | Disorder | 195967001 | Asthma |
|  |  |  |  | Disorder | 13645005 | Chronic obstructive pulmonary  disease |
|  |  |  |  | Disorder | 36599006 | Chronic fibrosis of lung |
|  |  |  |  | Disorder | 233703007 | Interstitial lung disease |
|  |  |  |  | Disorder | 190905008 | Cystic fibrosis |
|  |  |  |  |  |  |  |
|  | Disorder | 85828009 | Autoimmune disease |  |  |  |
|  |  |  |  | Disorder | 69896004 | Rheumatoid arthritis |
|  |  |  |  | Disorder | 200936003 | Lupus erythematosus |
|  |  |  |  | Disorder | 24526004 | Inflammatory bowel disease |
|  |  |  |  | Disorder | 24700007 | Multiple sclerosis |
|  |  |  |  |  |  |  |

***Table 3.* SNOMED CT concepts for risk factors from COVID-19 surveillance ontology.**

|  | **Semantic tag** | **ConceptID** | **Term** |
| --- | --- | --- | --- |
| **Exposure** |  |  |  |
|  | Event | 1240431000000104 | Exposure to SARS-CoV-2 (severe acute respiratory syndrome  coronavirus 2) infection |
|  | Event | 1240441000000108 | Close exposure to severe acute respiratory syndrome  coronavirus 2 infection (event) |
| **Foreign travel** |  |  |  |
|  | Observable entity | 161085007 | Detail of history of foreign travel |
|  | Observable entity | 810811000000107 | Date of foreign travel |
|  | Clinical finding | 506931000000109 | Recent travel to disease affected area |
|  | Observable entity | 161086008 | Recent countries visited |
|  | Observable entity | 161086008 | Recent countries visited |
|  |  |  |  |

***Table 4.* SNOMED CT concepts for investigations from COVID-19 surveillance ontology.**

|  | **Semantic tag** | **ConceptID** | **Term** |
| --- | --- | --- | --- |
| **Virology** |  |  |  |
|  | Procedure | 1240511000000106 | Detection of SARS-CoV-2 (severe acute respiratory syndrome  coronavirus 2) using polymerase chain reaction technique |
|  | Procedure | 1320971000000102 | Taking of swab for SARS-CoV-2 (severe acute respiratory  syndrome coronavirus 2) |
|  | Situation | 1321031000000105 | Self-taken swab for SARS-CoV-2 (severe acute respiratory  syndrome coronavirus 2) completed |
|  | Situation | 1321041000000101 | Self-taken swab for SARS-CoV-2 (severe acute respiratory  syndrome coronavirus 2) offered |
|  | Situation | 1321051000000103 | Swab for SARS-CoV-2 (severe acute respiratory  syndrome coronavirus 2) taken by healthcare professional |
|  | Procedure | 1240471000000102 | Measurement of severe acute respiratory syndrome  coronavirus 2 antigen |
| **Serology** |  |  |  |
|  | Procedure | 1240461000000109 | Measurement of SARS-CoV-2 (severe acute respiratory  syndrome coronavirus 2) antibody |
|  |  |  |  |

***Table 5.* SNOMED CT concepts for COVID-19 status from COVID-19 surveillance ontology – with allocation to terms of certainty of diagnosis.**

|  | **Semantic tag** | **ConceptID** | **Term** |
| --- | --- | --- | --- |
| **Definite** |  |  |  |
|  | Situation | 1300721000000109 | COVID-19 confirmed by laboratory test |
|  | Clinical finding | 1240581000000104 | SARS-CoV-2 (severe acute respiratory syndrome coronavirus  2) detected |
|  | Substance | 1240391000000107 | Antigen of severe acute respiratory syndrome coronavirus 2 |
|  | Substance | 1240411000000107 | Ribonucleic acid of SARS-CoV-2 (severe acute respiratory syndrome coronavirus) 2 |
| **Probable** |  |  |  |
|  | Disorder | 1240751000000100 | COVID-19 |
|  | Situation | 1300731000000106 | COVID-19 confirmed using clinical diagnostic criteria |
|  | Disorder | 1240561000000108 | Encephalopathy caused by SARS-CoV-2 (severe acute  respiratory syndrome coronavirus 2) |
|  | Disorder | 1240571000000101 | Gastroenteritis caused by SARS-CoV-2 (severe acute  respiratory syndrome coronavirus 2) |
|  | Disorder | 1240531000000103 | Myocarditis caused by SARS-CoV-2 (severe acute respiratory  syndrome coronavirus 2) |
|  | Disorder | 1240521000000100 | Otitis media caused by SARS-CoV-2 (severe acute respiratory  syndrome coronavirus 2) |
|  | Disorder | 1240551000000105 | Pneumonia caused by SARS-CoV-2 (severe acute respiratory  syndrome coronavirus 2) |
|  | Disorder | 1240541000000107 | Upper respiratory tract infection caused by SARS-CoV-2 (severe acute respiratory syndrome coronavirus 2) |
|  | Disorder | 186747009 | Coronavirus infection |
| **Possible** |  |  |  |
|  | Situation | 1240761000000102 | Suspected COVID-19 |
|  | Procedure | 1240451000000106 | Telephone consultation for suspected SARS-CoV-2 (severe acute  respiratory syndrome coronavirus 2) |
|  | Situation | 700217006 | Suspected coronavirus infection |
|  | Event | 1240431000000104 | Exposure to SARS-CoV-2 (severe acute respiratory syndrome coronavirus 2) infection |
|  | Event | 1240441000000108 | Close exposure to SARS-CoV-2 (severe acute respiratory syndrome coronavirus 2) infection |
|  | Procedure | 1240471000000102 | Measurement of SARS-CoV-2 (severe acute respiratory syndrome coronavirus 2) antigen |
|  | Procedure | 1240511000000106 | Detection of SARS-CoV-2 (severe acute respiratory syndrome coronavirus 2) using polymerase chain reaction technique |
|  | Procedure | 1320971000000102 | Taking of swab for SARS-CoV-2 (severe acute respiratory syndrome coronavirus 2) |
|  | Situation | 1321031000000105 | Self-taken swab for SARS-CoV-2 (severe acute respiratory syndrome coronavirus 2) completed |
|  | Situation | 1321041000000101 | Self-taken swab for SARS-CoV-2 (severe acute respiratory syndrome coronavirus 2) offered |
|  | Situation | 1321051000000103 | Swab for SARS-CoV-2 (severe acute respiratory syndrome coronavirus 2) taken by healthcare professional |
|  | Procedure | 1240461000000109 | Measurement of SARS-CoV-2 (severe acute respiratory syndrome coronavirus 2) antibody |
|  | Observable entity | 1008541000000105 | Coronavirus ribonucleic acid detection assay |
|  | Observable entity | 1029481000000103 | Coronavirus nucleic acid detection assay |
|  | Qualifier value | 1240421000000101 | Serotype SARS-CoV-2 (severe acute respiratory syndrome coronavirus 2) |
|  | Substance | 1240401000000105 | Antibody to SARS-CoV-2 (severe acute respiratory syndrome coronavirus 2) |
| **Excluded** |  |  |  |
|  | Situation | 1321101000000103 | COVID-19 excluded |
|  | Situation | 1321111000000101 | COVID-19 excluded by laboratory test |
|  | Situation | 1321121000000107 | COVID-19 excluded using clinical diagnostic criteria |
|  | Clinical finding | 1240591000000102 | SARS-CoV-2 (severe acute respiratory syndrome coronavirus 2) not  detected |
|  |  |  |  |

***Table 6.* SNOMED CT concepts for interventions from COVID-19 surveillance ontology.**

|  | **Semantic tag** | **ConceptID** | **Term** | |
| --- | --- | --- | --- | --- |
| **Vaccination** |  |  | |  |
|  | Procedure | 1240491000000103 | | SARS-CoV-2 (severe acute respiratory syndrome coronavirus 2) vaccination |
|  | Situation | 1240681000000103 | | SARS-CoV-2 (severe acute respiratory syndrome coronavirus 2)  vaccination not done |
|  | Situation | 1240661000000107 | | SARS-CoV-2 (severe acute respiratory syndrome coronavirus 2) vaccination contraindicated |
|  | Situation | 1240671000000100 | | SARS-CoV-2 (severe acute respiratory syndrome coronavirus 2)  vaccination not indicated |
|  | Situation | 1240651000000109 | | SARS-CoV-2 (severe acute respiratory syndrome coronavirus 2) vaccination declined |
|  | Clinical finding | 1240631000000102 | | Did not attend SARS-CoV-2 (severe acute respiratory syndrome  coronavirus 2) vaccination |
|  | Situation | 1240701000000101 | | SARS-CoV-2 (severe acute respiratory syndrome coronavirus 2) vaccine not available |
|  | Clinical finding | 1240601000000108 | | High priority for SARS-CoV-2 (severe acute respiratory syndrome  coronavirus 2) vaccination |
|  |  |  |  | |

***Table 7.* SNOMED CT concepts for processes of care from COVID-19 surveillance ontology.**

|  | **Semantic tag** | **ConceptID** | **Term** |
| --- | --- | --- | --- |
| **COVID-19** |  |  |  |
| **encounter** | Situation | 1240731000000107 | Advice given about SARS-CoV-2 (severe acute respiratory syndrome coronavirus 2) by telephone |
|  | Situation | 1240721000000105 | Advice given about SARS-CoV-2 (severe acute respiratory syndrome coronavirus 2) infection |
|  | Situation | 1240711000000104 | Educated about SARS-CoV-2 (severe acute respiratory syndrome coronavirus 2) infection |
|  | Procedure | 710874007 | Education about cross infection prevention |
|  | Procedure | 737612005 | Education about isolation for infection control |
|  | Situation | 1321171000000106 | Provision of advice, assessment or treatment limited due  to COVID-19 pandemic |
|  | Situation | 1321221000000103 | Consultation via video conference not available |
|  | Procedure | 1240451000000106 | Telephone consultation for suspected SARS-CoV-2 (severe acute respiratory syndrome coronavirus 2) |
| **Isolation** |  |  |  |
|  | Procedure | 170497006 | Quarantine |
|  | Procedure | 170499009 | Isolation of infection contact |
|  | Procedure | 170500000 | Isolation of infection carrier |
|  | Procedure | 361235007 | Isolation of infected patient |
|  | Regime | 370835007 | Monitoring for signs and symptoms of infection |
|  | Regime | 444908001 | Isolation nursing in negative pressure isolation  environment |
|  | Procedure | 742879000 | Management of isolation for infection control |
|  | Regime | 9478004 | Prospective focused infection control surveillance |
|  | Finding | 422650009 | Social isolation |
|  | Procedure | 1321131000000109 | Self-isolation to prevent exposure of community  to contagion |
|  | Procedure | 1321161000000104 | Household isolation to prevent exposure of community  to contagion |
|  | Procedure | 1321151000000102 | Shielding of uninfected subject to prevent exposure to  contagion |
|  | Procedure | 1321141000000100 | Shielding of household to prevent exposure of uninfected subject  to contagion |
|  | Procedure | 1321231000000101 | Signposting to CHMS (COVID-19 Home Management Service) |
|  | Procedure | 1321061000000100 | Signposting to NHS online isolation note service |
|  | Finding | 1321071000000107 | Has NHS digital isolation note |
|  | Finding | 1321091000000106 | Household isolation note issued to patient |
|  | Finding | 1321081000000109 | Self-isolation note issued to patient |
| **Contact** |  |  |  |
| **tracing** | Regime | 170503003 | Surveillance of contact |
|  | Procedure | 225368008 | Contact tracing |
|  | Clinical finding | 305559001 | Under care of contact tracing nurse |
|  | Clinical finding | 305736005 | Seen by contact tracing nurse |
|  | Procedure | 306030003 | Referral by contact tracing nurse |
|  | Procedure | 306323004 | Referral to contact tracing nurse |
|  | Procedure | 306497009 | Discharge by contact tracing nurse |
|  |  |  |  |

***Table 8.* SNOMED CT concepts for outcomes from COVID-19 surveillance ontology.**

|  | **Semantic tag** | **ConceptID** | **Term** |
| --- | --- | --- | --- |
| **Hospitalisation** |  |  |  |
|  | Procedure | 32485007 | Hospital admission |
|  | Record artefact | 24651000000105 | Hospital admission note |
|  | Procedure | 183452005 | Emergency hospital admission |
| **Oxygen** |  |  |  |
| **therapy** | Procedure | 410204009 | Oxygen therapy management |
|  | Finding | 371825009 | Patient on oxygen |
|  | Finding | 931000119107 | Dependence on supplemental oxygen |
|  | Regime | 707808001 | Oxygen therapy support |
|  | Procedure | 426990007 | Home oxygen therapy |
|  | Procedure | 371907003 | Oxygen administration by nasal cannula |
|  | Finding | 794131000000104 | Oxygen nasal cannula in situ |
| **Intensive care** |  |  |  |
|  | Procedure | 305351004 | Admission to intensive care unit |
|  | Regime | 182810003 | Intensive care monitoring |
|  | Procedure | 305351004 | Admission to intensive care unit |
|  | Record artefact | 959831000000105 | Adult intensive care plan |
| **Mechanical** |  |  |  |
| **ventilation** | Procedure | 266700009 | Assisted breathing |
|  | Procedure | 40617009 | Artificial respiration |
|  | Regime | 47545007 | Continuous positive airway pressure  ventilation treatment |
|  | Procedure | 226471000000101 | Invasive ventilation |
|  | Procedure | 447837008 | Noninvasive positive pressure ventilation |
|  | Procedure | 428311008 | Noninvasive ventilation |
|  | Procedure | 233573008 | Extracorporeal membrane oxygenation |
|  | Procedure | 82433009 | Continuous negative pressure ventilation  treatment |
|  | Qualifier value | 707765006 | On ventilator |
| **Discharge** |  |  |  |
|  | Procedure | 308283009 | Discharge from hospital |
|  | Observable entity | 183797002 | Duration of inpatient stay |
| **Death** |  |  |  |
|  | Event | 419620001 | Death |
|  | Event | 26636000 | Sudden death |
|  | Event | 59283008 | Maternal death |
|  | Event | 16983000 | Death in hospital |
|  | Finding | 184293009 | Patient died at home |
|  | Finding | 184297005 | Patient died in hospital |
|  | Finding | 184295002 | Patient died in nursing home |
|  | Finding | 698747008 | Patient died in care home |
|  |  |  |  |

**S3 – Delphi panel and complete questions from Round 2.**

***Table 9.* Delphi Panel Members**

| **Name** | **Discipline** | **Location** |
| --- | --- | --- |
| Amanda Terry | General Practice & Clinical Informatics | Western University, Canada |
| Bhautesh Jani | General Practice | University of Glasgow, Scotland. |
| Craig Kuziemsky | Clinical Informatics | MacEwan University, Canada. |
| Frances Mair | General Practice | University of Glasgow, Scotland. |
| Jorgen Bauwens | Clinical Informatics | University of Basel, Switzerland. |
| Oscar Tamburis | Clinical Informatics | University of Naples Federico II, Italy. |
| Richard Schreiber | Clinical Informatics | Geisinger Commonwealth School of Medicine, USA. |
| Siaw-Teng Liaw | General Practice & Clinical Informatics | University of New South Wales, Australia. |
| Thomas Freeman | General Practice | Western University, Canada. |
| Tom Fahey | General Practice | RCSI, Ireland. |

**Supplementary questionnaire from Delphi Consensus Round 2.**

The concepts of the COVID-19 ontology are organised across 8 high level concepts. Please indicate your level of agreement with the coverage of concepts given under each heading.


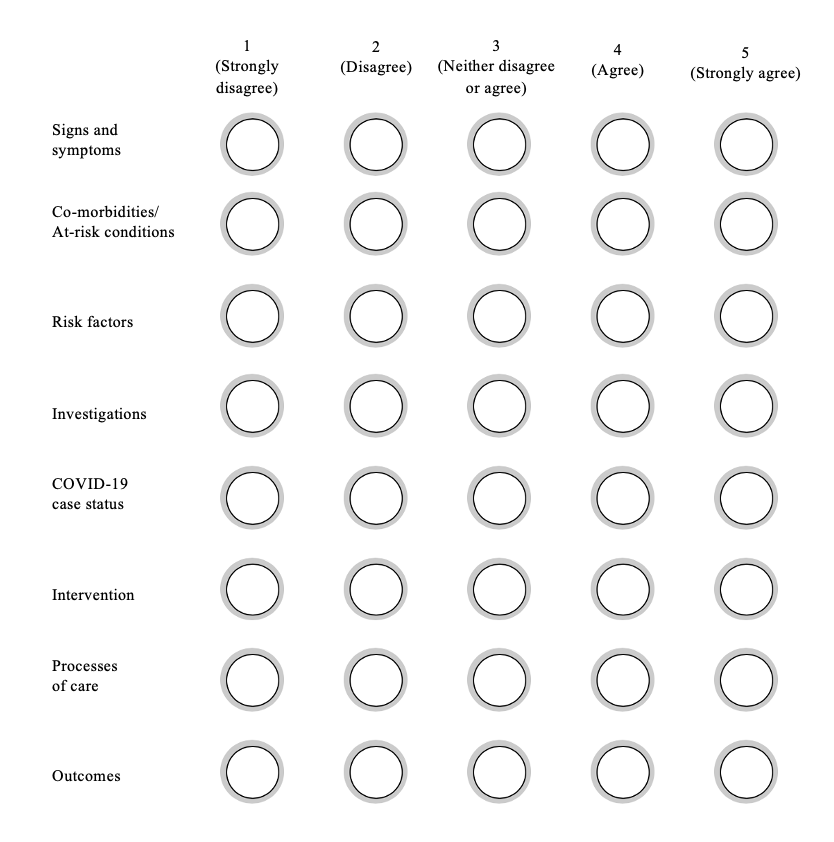


The COVID-19 ontology in its current format is suitable for case ascertainment of COVID-19 in my local primary care setting.


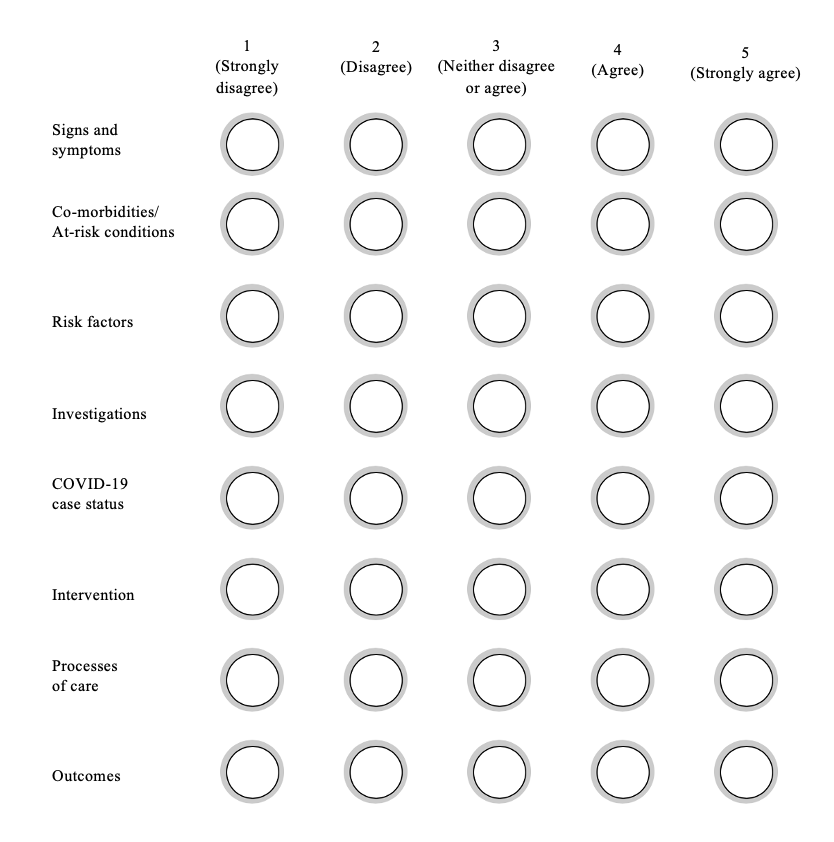


Coding clinical outcomes such as hospitalisation, oxygen therapy, ICU admission and mortality is an essential component of any COVID-19 surveillance ontology.


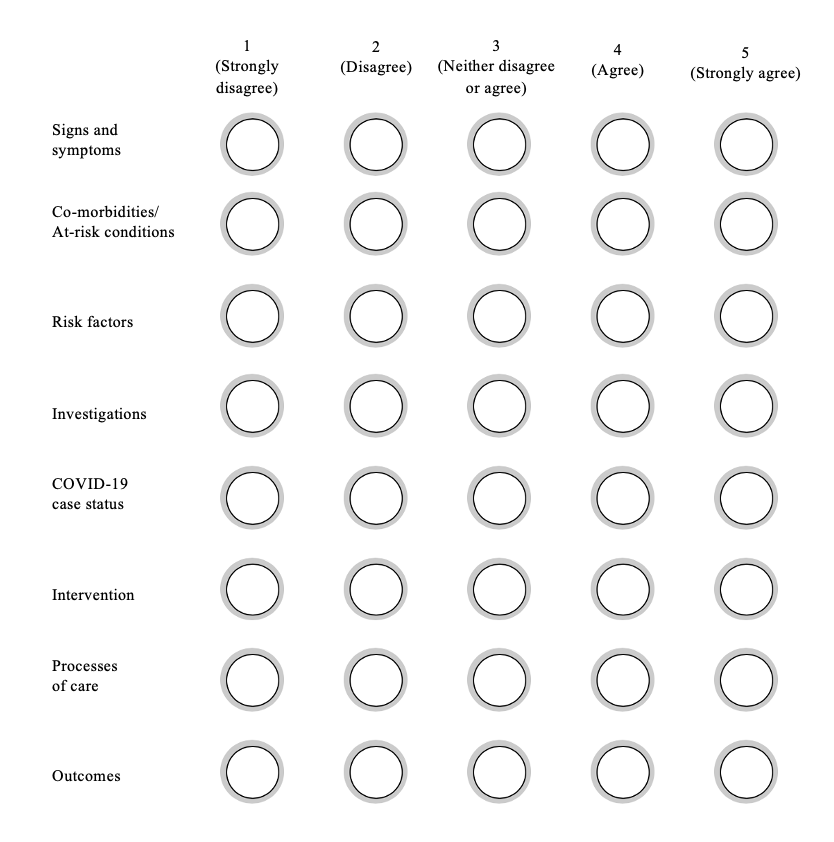


Are there any aspects you foresee being relevant to COVID-19 surveillance and clinical care in the future which may need adding to the current ontology?

Your answer:

What type of clinical coding system does your local health care system use?

Your answer:
